# Comparing recruitment strategies in a study of acupuncture for chronic back pain

**DOI:** 10.1186/1471-2288-9-69

**Published:** 2009-10-27

**Authors:** Karen J Sherman, Rene J Hawkes, Laura Ichikawa, Daniel C Cherkin, Richard A Deyo, Andrew L Avins, Partap S Khalsa

**Affiliations:** 1 Group Health Research Institute, 1730 Minor Avenue, Suite 1600, Seattle, WA 98101, USA; 2 Departments of Family Medicine and Internal Medicine, Oregon Health and Science University, 3181 SW Sam Jackson Park Rd, Portland, OR 97239, USA; 3 Division of Research, Northern California Kaiser-Permanente, 2000 Broadway, Oakland, CA 94612, USA; 4 Division of Extramural Research and Training, National Center for Complementary and Alternative Medicine, National Institutes of Health, 6707 Democracy Boulevard, Suite 401, Bethesda, MD 20892, USA

## Abstract

**Background:**

Meeting recruitment goals is challenging for many clinical trials conducted in primary care populations. Little is known about how the use of different recruitment strategies affects the types of individuals choosing to participate or the conclusions of the study.

**Methods:**

A secondary analysis was performed using data from participants recruited to a clinical trial evaluating acupuncture for chronic back pain among primary care patients in a large integrated health care organization. We used two recruitment methods: mailed letters of invitation and an advertisement in the health plan's magazine. For these two recruitment methods, we compared recruitment success (% randomized, treatment completers, drop outs and losses to follow-up), participant characteristics, and primary clinical outcomes. A linear regression model was used to test for interaction between treatment group and recruitment method.

**Results:**

Participants recruited via mailed letters closely resembled those responding to the advertisement in terms of demographic characteristics, most aspects of their back pain history and current episode and beliefs and expectations about acupuncture. No interaction between method of recruitment and treatment group was seen, suggesting that study outcomes were not affected by recruitment strategy.

**Conclusion:**

In this trial, the two recruitment strategies yielded similar estimates of treatment effectiveness. However, because this finding may not apply to other recruitment strategies or trial circumstances, trials employing multiple recruitment strategies should evaluate the effect of recruitment strategy on outcome.

**Trial registration:**

Clinical Trials.gov NCT00065585.

## Background

Clinical studies often encounter difficulties in recruiting participants, leading to underpowered studies that fall short of targeted sample sizes [1]. When attempting to augment recruitment to achieve sample size targets, researchers can employ a variety of recruitment strategies [2]. However, different recruitment strategies could conceivably lead to different conclusions about treatment efficacy [3]. This might be particularly true when participants are not masked to treatment and have strong beliefs about the usefulness of the therapy. For example, in studies of complementary and alternative medicine, different populations might have vastly different pre-conceived notions about such treatments and these differences could affect a trial's estimates of treatment effects. Conceivably, persons recruited via advertisements might be more enthusiastic about complementary and alternative medicine and thus, studies using such volunteers might demonstrate greater benefits. Therefore, whenever possible, it is important to determine whether study outcomes are affected by recruitment strategy.

As part of a large trial of acupuncture in two integrated healthcare systems [4], we had the opportunity to use multiple recruitment strategies in one of the study locations. In this report, we compare the effects of two different recruitment strategies in terms of their efficiency, the characteristics of the patients who respond, and their responses to treatment.

## Methods

This study analyzed data collected for a large two-site randomized controlled trial evaluating acupuncture for chronic low back pain. The trial was approved by the institutional review boards for both sites (Group Health in Seattle and Kaiser Permanente in Northern California) where the study was conducted. Participants were individuals with non-specific chronic low back pain of at least three months duration, but who lacked symptoms of sciatica and who had never tried acupuncture for any reason. They were recruited between March 2004 and August 2006 and were randomized to one of three types of acupuncture (individualized, standardized, or simulated acupuncture) or usual care and all four treatments were supplemented with a self-care book. In this trial, participants who received acupuncture or simulated acupuncture had greater improvements in functional status and symptoms at the end of the treatment and at follow-up than those receiving usual medical care. The trial design and primary findings are reported in detail elsewhere [4,5], so only a brief summary is provided here. Participants assigned to acupuncture (or simulated acupuncture) received 10 treatments over 7 weeks (twice weekly for 3 weeks and weekly for 4 weeks). Telephone interviewers masked to treatment conducted follow-up interviews at 8, 26, and 52 weeks. The primary outcomes were back-related functional status and symptom bothersomeness. At 8 weeks, back symptoms and function had improved in all acupuncture or simulated acupuncture groups. The positive impact of the acupuncture treatments on function was still evident at 52 weeks.

In order to reach our targeted sample size in the Seattle site, we tried a variety of recruitment strategies. Almost 90% of study participants were recruited through mailed letters and advertisements in the health plan magazine. In previous studies, we had largely recruited using mailed letters and thus, were interested in knowing whether the individuals recruited through advertisements were similar to those recruited by mailed letters in terms of their sociodemographic and back pain characteristics, their beliefs about acupuncture, and their treatment outcomes. The remaining 10% of Seattle study participants were recruited by clinic fliers, the study website, or by referral from someone who became aware of the study. Those individuals were excluded from this analysis because there were so few of them. We also excluded individuals recruited at the Oakland site from this analysis because all but 30 of the 287 Oakland participants were recruited by means of mailed letters to persons having recently receiving care for back pain.

### Recruitment Strategies

#### Recruitment by mail

Using automated visit data, members of Group Health in Seattle whose visits to healthcare providers resulted in diagnoses consistent with non-specific low back pain were identified. Three to 12 months after their visit, potential participants were mailed a letter that explained the study, described eligibility requirements, and invited participation. If interested in participating, members returned a signed statement indicating their willingness to be contacted by study staff. An interviewer phoned those members to answer questions and determine eligibility using a computer-assisted screening program. Eligible members were guided through the consent process by an interviewer who then sent a copy of the consent form for them to sign and return. Once written consent was obtained, another interviewer contacted the potential participant to administer the baseline questionnaire. If still willing to participate, participants were randomized to one of four groups (two types of acupuncture, simulated acupuncture or usual care). If the participant was randomized to acupuncture or simulated acupuncture, the interviewer scheduled the first two acupuncture appointments.

#### Recruitment by advertisement in health plan magazine

Advertisements were placed in the health plan's quarterly magazine on five occasions at three to six month intervals between October 2004 and April 2006. The advertisement instructed interested persons to call the toll-free number and leave their name and phone number. All prospective participants were telephoned by an interviewer, any questions were answered and if appropriate, their eligibility was determined with the assistance of a computer-screening program. If eligible, the member was guided through the consent and baseline interview process using the same procedures described above.

### Outcome Measures

#### Treatment completion and loss to follow-up

The number of completed treatments (characterized in this report as none, 1 to 7, or 8 to 10 for the acupuncture and simulated acupuncture groups), with 8 to 10 treatments representing a full course of treatment, was obtained. In addition, formal treatment drop-outs and those withdrawing from follow-up were also assessed.

#### Baseline characteristics

Demographic variables, back pain history and characteristics of current episode, satisfaction with back pain care, current use of medication and exercise for back pain, and knowledge and expectations of acupuncture as well as preferred treatment for back pain were collected at baseline.

#### Follow-up outcomes

At 8, 26, and 52 weeks, telephone interviews were conducted by persons unaware of the participant's treatment assignment. For this manuscript, we present data for the primary treatment outcomes, back-related functional status and symptom bothersomeness by recruitment source. The Roland Morris Disability Questionnaire (RMDQ), which is reliable, valid and appropriate for telephone administration, was used to measure back-related functional status [6]. Symptom bothersomenenss, which is highly correlated with pain intensity in our data (r = 0.80, p < 0.0001), was measured by asking participants to rate how bothersome their pain had been during the past week on a 0 ("not at all bothersome") to 10 ("extremely bothersome") scale.

### Statistical Analyses

Participant flow was reported as frequencies and percentages. Baseline characteristics were displayed as means (and standard deviations) or percentages. Because we did not find differences in outcomes between the acupuncture and simulated acupuncture groups, results are presented comparing any acupuncture or simulated acupuncture treatment with usual care. For simplicity, the acupuncture and simulated acupuncture groups are subsequently referred to as the "acupuncture group". When testing for differences between sources of recruitment, we used t-tests for continuous variables (means), Wilcoxon non-parametric tests for differences in ranks (medians), and chi square tests or Fisher exact tests for categorical variables. A linear regression model was used to examine whether or not the association between treatment group and primary outcomes differed by recruitment method. The model included main effects for treatment group and recruitment method and an interaction between these two to test for effect modification. The model was repeated at each follow-up time point and for each primary outcome: RMDQ and symptom bothersomeness. Including educational status and back pain duration in these models did not change the outcomes, so results are based on models without additional covariates. We used SAS/STAT Version 9.1 [7] and all tests of significance were two-sided.

## Results

### Response Rates by Recruitment Strategy

We received responses from 1269 individuals, of whom 859 responded to invitation letters and 410 responded to a magazine advertisement (Table 1). Only 13 persons who responded to the magazine advertisement had previously been mailed an invitation letter.

**Table 1:** Study recruitment resolution by recruitment method and randomization group

	Mailed Letter		Magazine Advertisement	
No. of responses received	859		410	
	N	%	N	%
Ineligible	597	69	270	66
Refused	36	4	14	3
Unable to contact	15	2	4	1
Recruitment ended before eligibility asessed	15	2	6	1
No. randomized	196	23	116	28
		
	Acupuncture	Usual care	Acupuncture	Usual care
		
	(N = 150)	(N = 46)	(N = 85)	(N = 31)
No. treatments received	N (%)		N (%)	
0	5 (3)	N/A	4 (5)	N/A
1-7	11 (7)	N/A	6 (7)	N/A
8-10	134 (89)	N/A	75 (88)	N/A
				
No. of withdrawals				
- no follow-up	3 (2)	3	3 (4)	0
- with follow-up	9 (6)	N/A	6 (7)	N/A

Slightly more of those responding to the magazine advertisements were ultimately enrolled in the study (28.2% of magazine responders versus 22.8% of letter invitees; p = 0.03). However, the proportion of persons randomized to acupuncture who completed at least 8 of the 10 treatments was virtually identical for both recruitment methods (88% vs. 89%; p = 0.96). Eight percent (12 of 150) of the persons in the acupuncture group recruited by mail withdrew from treatment early vs. 10.6% (9 of 85) of those responding to the magazine advertisements (p = 0.49). Most of the persons withdrawing from treatment agreed to telephone follow-up interviews (75% of letter invitees who withdrew vs. 67% of magazine advertisement enrollees who withdrew; p = 1.0). Three of the 46 persons in the usual care group recruited by invitation letters withdrew from the study versus none of 31 in the magazine advertisement group (p = 0.27).

### Baseline Characteristics by Recruitment Strategy

Tables 2 and 3 present the baseline characteristics of persons by randomized treatment group and recruitment strategy. Among demographic characteristics, only educational status differed for the two methods of recruitment. Those responding to the health plan magazine advertisements were more likely to be college graduates (p = 0.01).

**Table 2:** Demographic and back pain baseline characteristics of study population by randomization group and recruitment method

	Acupuncture		Usual care		Total		
	Mailed	Magazine	Mailed	Magazine	Mailed	Magazine	
	Letter	Ad	Letter	Ad	Letter	Ad	p for
	(n = 150)	(n = 85)	(n = 46)	(n = 31)	(n = 196)	(n = 116)	difference*
**Demographic characteristics**							

Age, mean(SD) years	46 (13)	49 (13)	45 (12)	49 (13)	46 (13)	49 (13)	0.07
Female, %	61	60	61	71	61	63	0.70
White, %	89	93	89	84	89	91	0.73
Hispanic origin, %	4	4	7	0	5	3	0.37
College graduate %	52	71	50	58	52	67	**0.01**
Married, %	63	55	63	65	63	58	0.38
Household income $45,000+/year, %	66	62	76	74	73	67	0.26
Employed, %	81	78	83	71	81	76	0.27

**Back Pain characteristics**							

Duration of low back pain at least one year, %	68	58	80	61	71	59	**0.03**
Prior surgery, hospitalization or injections, %	13	8	11	19	12	11	0.78
Reduced activity for 7+ days in last 3 months due to low back pain, %	29	34	30	32	29	34	0.42
Days of pain in last 3 months:							
mean (SD) number of days	68 (26)	72 (21)	72 (25)	78 (21)	69 (25)	74 (21)	0.43
median number of days	80	80	90	90	80	83	
Pain below knee %	17	18	17	23	17	19	0.63
Symptom bothersomeness, mean (SD) (0-10 scale)	5.1 (2.2)	4.9 (2.3)	5.4 (2.0)	5.7 (2.2)	5.1 (2.1)	5.1 (2.3)	0.98
RMDQ, mean (SD) (0-23 scale)	9.9 (5.1)	9.5 (4.9)	10.6 (4.8)	10.0 (5.1)	10.1 (5.0)	9.6 (5.0)	0.45
Expectation of at least moderate improvement in low back pain in next year, %	45	44	50	52	46	46	0.99

* p < 0.05 is indicated in bold typeface

Abbreviation: RMDQ, Roland Morris Disability Questionnaire

**Table 3:** Additional baseline characteristics of study population by randomization group and recruitment method

	Acupuncture		Usual care		Total		
	Mailed	Magazine	Mailed	Magazine	Mailed	Magazine	
	Letter	Ad	Letter	Ad	Letter	Ad	p for
	(n = 150)	(n = 85)	(n = 46)	(n = 31)	(n = 196)	(n = 116)	difference*
**Back pain: satisfaction with prior care and some concurrent treatments**							

Medication use in past week, %	67	64	72	65	68	64	0.43
Satisfaction with care: overall, %							**0.002**
Very or somewhat satisfied	45	32	39	39	43	34	
Not satisfied or dissatisfied	51	53	59	52	53	53	
Missing	4	15	2	10	4	14	
At least moderately agree will try to manage back pain by self in future, %	23	20	17	23	22	21	0.80
Back exercise in past week:							
% any	66	69	59	71	64	70	0.32
mean (SD) number of days	2.5 (2.4)	2.5 (2.4)	2.3 (2.4)	3.0 (2.6)	2.5 (2.4)	2.6 (2.4)	0.47
median number of days	2.0	2.0	2.0	3.0	2.0	2.0	
Active exercise in past week:							
% any	73	84	80	74	74	81	0.18
mean (SD) number of days	2.6 (2.2)	3.3 (2.2)	2.8 (1.9)	2.8 (2.3)	2.6 (2.1)	3.2 (2.2)	**0.03**
median number of days	2.0	3.0	3.0	2.0	3.0	3.0	

**Treatment expectations, preferences, and knowledge**							

Expectation of helpfulness of acupuncture, mean (SD) (0-10 scale) (based on non-missing data)	6.3 (1.8)	6.6 (1.8)	7.0 (1.7)	6.9 (1.8)	6.5 (1.8)	6.6 (1.8)	0.44
Preferred treatment, %							0.29
Acupuncture	25	37	33	36	28	38	
Other CAM	43	38	33	32	42	38	
Conventional	25	20	33	26	28	23	
Other/Unknown	7	6	2	7	1	2	
Any knowledge of acupuncture, %	32	38	33	48	32	41	0.13
Told about acupuncture effectiveness, %							0.33
Very effective	27	34	30	23	28	31	
Less than very effective	32	31	22	42	30	34	
Unknown	41	35	48	36	42	35	
Impression of acupuncture, %							0.17
Very positive	21	25	22	23	21	24	
Moderately positive	28	42	44	32	32	40	
Negative or Slightly positive	51	33	35	45	47	36	

* p < 0.05 is indicated in bold typeface

Overall, history of back pain and the intensity of the current episode, including both primary outcome measures, were reassuringly similar for the two recruitment strategies. However, a somewhat higher proportion of persons recruited via mailed letters reported that their back pain had lasted at least a year (p = 0.03). In addition, persons recruited via mailed letters were more likely to report being "very or somewhat satisfied" with the overall care they had received for back pain in the past (Table 3, p, = 0.002, respectively). Finally, persons recruited via mailed letters exercised slightly fewer days per week (Table 3, p = 0.03).

Expectation of acupuncture's helpfulness, preferred treatment for back pain, knowledge of acupuncture, previous information about acupuncture's effectiveness, and impression of acupuncture did not differ substantially by recruitment method.

### Primary Outcomes by Recruitment Strategy

Table 4 displays change scores for symptom bothersomeness and functional disability (RMDQ) in the acupuncture and usual care groups by recruitment method at the three follow-up periods. At all three time points and for both primary outcomes, the interaction between treatment group and recruitment method was not statistically significant. This demonstrates that outcomes by treatment group were similar for both recruitment strategies.

**Table 4:** Unadjusted mean difference from baseline to follow-up for outcomes by treatment group and recruitment method

	Acupuncture				Usual care				Test for interaction of treatment group and recruitment method
	
Follow-up Interval Outcome		Mailed Letter	Magazine Advertisement			Mailed Letter	Magazine Advertisement		
		Mean		Mean		Mean		Mean	
	N	(95% CI)	N	(95% CI)	N	(95% CI)	N	(95% CI)	
**8-week**									
Symptom bothersomeness	144	-1.8 (-2.3, -1.4)	81	-1.6 (-2.2, -1.0)	41	-0.8 (-1.5, -0.1)	30	-1.5 (-2.5, -.05)	0.82
RMDQ	144	-3.8 (-4.7, -2.9)	82	-3.3 (-4.4, -2.1)	41	-0.9 (-2.3, +0.5)	30	-2.3 (-3.7, -1.0)	0.99
									
**26-week**									
Symptom bothersomeness	143	-1.3 (-1.8, -0.8)	82	-1.6 (-2.3, -1.0)	40	-1.4 (-2.2, -0.5)	29	-1.4 (-2.5, -0.3)	0.30
RMDQ	143	-3.5 (-4.4, -2.5)	82	-3.8 (-4.8, -2.8)	40	-2.5 (-4.1, -1.0)	29	-2.5 (-4.2, -0.7)	0.25
									
**52-week**									
Symptom bothersomeness	140	-1.4 (-1.9, -0.9)	80	-1.8 (-2.4, -1.1)	38	-1.5 (-2.4, -0.6)	30	-1.3 (-2.2, -0.3)	0.20
RMDQ	140	-3.5 (-4.4, -2.7)	80	-4.1 (-5.1, -3.1)	38	-3.2 (-4.7, -1.8)	30	-2.4 (-4.4, -0.4)	0.17

Note: main effect of recruitment method is not significant in any of the models

Abbreviation: RMDQ, Roland Morris Disability Questionnaire

## Discussion

Our estimates of the benefit of acupuncture for chronic low back pain were not affected by recruitment strategy. Sociodemographic characteristics, back pain history and past treatments, and perceptions and expectations about their back pain and about acupuncture were also unaffected by recruitment strategy.

A previous study of transcutaneous electrical nerve stimulation for chronic back pain compared the characteristics of persons recruited via media advertisements with regular patients from a pain clinic [8]. This study found substantial differences between the populations recruited using these different methods in back pain history and characteristics, employment status, and improvement over a 4 to 6 week period. They recommended that researchers pay careful attention to the method of recruitment so that the readers can understand how to apply the results of their findings.

Our study participants had never had acupuncture before and likely resembled primary care patients who fail to obtain adequate relief for their back pain from conventional treatments. In fact, cohort studies in other primary care populations have found similar levels of dysfunction among primary care consulters for low back pain [9,10]. These data suggest that our methods of recruitment resulted in a study population whose outcomes may be broadly applicable to a variety of primary care populations.

Strengths of this study included a comprehensive battery of baseline questions and a similar procedure for enrollment in the study regardless of recruitment strategy. However, because we could not distinguish between acute and chronic back pain using diagnoses included in electronic medical records, we cannot estimate how representative the responders to mailed invitations were to the group of persons who sought care for chronic back pain, for example by comparison to medical records of persons with chronic back pain. In addition, because our patients were recruited from an integrated health care system, we do not know how representative they would be of all primary care patients seeking care for chronic back pain.

Although our finding that recruitment method did not influence study outcomes in this acupuncture trial was reassuring, we cannot be certain that this would be true for other types of complementary medicine. We recommend that other studies using multiple strategies for recruitment evaluate whether these impact the study outcomes.

## Conclusion

The characteristics of persons with chronic low back pain recruited for a trial of acupuncture using two different strategies were remarkably similar. Of most importance, the estimated benefits of acupuncture were similar for the two recruitment strategies. However, this may not always be the case and future trials should evaluate the importance of recruitment strategy on outcome.

## Competing interests

The authors declare that they have no competing interests.

## Authors' contributions

KJS and RJH led the author team in the conceptualization and design of the analysis involved in this report; RJH and LI conducted the analysis; all authors were involved in interpretation of the findings; KJS, DCC, LI and RJH drafted the manuscript and all authors read and approved the final manuscript.

## Pre-publication history

The pre-publication history for this paper can be accessed here:

http://www.biomedcentral.com/1471-2288/9/69/prepub
